# Generalized Baum-Welch Algorithm Based on the Similarity between Sequences

**DOI:** 10.1371/journal.pone.0080565

**Published:** 2013-12-20

**Authors:** Vahid Rezaei, Hamid Pezeshk, Horacio Pérez-Sa'nchez

**Affiliations:** 1 Department of Mathematics and Statistics, Faculty of Financial Science, University of Economic Sciences, Tehran, Iran; 2 School of Computer Science, Institute for Research in Fundamental Science (IPM), Tehran, Iran; 3 School of Mathematics, Statistics and Computer Science, University of Tehran, Iran; 4 Computer Science Department, Catholic University of Murcia (UCAM), Murcia, Spain; Université de Nantes, France

## Abstract

The profile hidden Markov model (PHMM) is widely used to assign the protein sequences to their respective families. A major limitation of a PHMM is the assumption that given states the observations (amino acids) are independent. To overcome this limitation, the dependency between amino acids in a multiple sequence alignment (MSA) which is the representative of a PHMM can be appended to the PHMM. Due to the fact that with a MSA, the sequences of amino acids are biologically related, the one-by-one dependency between two amino acids can be considered. In other words, based on the MSA, the dependency between an amino acid and its corresponding amino acid located above can be combined with the PHMM. For this purpose, the new emission probability matrix which considers the one-by-one dependencies between amino acids is constructed. The parameters of a PHMM are of two types; transition and emission probabilities which are usually estimated using an EM algorithm called the Baum-Welch algorithm. We have generalized the Baum-Welch algorithm using similarity emission matrix constructed by integrating the new emission probability matrix with the common emission probability matrix. Then, the performance of similarity emission is discussed by applying it to the top twenty protein families in the Pfam database. We show that using the similarity emission in the Baum-Welch algorithm significantly outperforms the common Baum-Welch algorithm in the task of assigning protein sequences to protein families.

## Introduction

Structure and function determination of newly discovered proteins, using the information contained in their amino acid sequences, is one of the most important problems in genomics [1]. Often, but certainly not always, as the homologous proteins have similar sequences and structures, they have similar functions [2]. The profile hidden Markov model (PHMM) can be applied to determine the related proteins by sequence comparison [3]. The parameters of a PHMM are of two types; transition and emission probabilities. Under a PHMM, there are two assumptions made for transition and emission probabilities as follows:

1. The 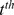 hidden state, given the 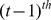 hidden state, is independent of previous states.
2. The 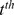 observation depends only on the 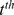 state.

The PHMM is specified as a triplet 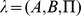 where 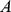 is the transition probability matrix, 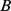 is the emission probability matrix and 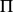 is the vector of initial probabilities. An important task in assigning a new protein sequence to a protein family is to estimate the parameters of the PHMM. The Parameters of a PHMM (transition probability matrix and emission probability matrix) can be estimated in two ways: they can be estimated either from the aligned sequences or unaligned sequences using the Baum-Welch algorithm [4].

The Baum-Welch algorithm works by guessing initial parameter values, then estimating the likelihood of the observation under the current parameters. This likelihood then will be used to re-estimate the parameters iteratively until a local maximum is reached. The Baum-Welch algorithm finds 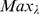 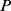
(observation 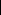 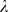) by considering only the information on the previous state of a hidden state. In other words, in the process of the Baum-Welch algorithm, it is assumed that given states the observations are independent and only the dependency between hidden states is considered. So, the dependency between observations can be combined with the PHMM. For this purpose, the multiple sequence alignment (MSA) which is a representative of a PHMM can be considered. In this paper the ClustalW program which is the current implementation of MSA is used for consideration of the dependency between observations.

Based on the MSA, one-by-one dependencies between corresponding amino acids of two current sequences that model the similarity between them can be appended to the PHMM. This approach in spirit is similar to the works proposed by Holmes [5], Qian and Goldstein [6] and Siepel [7] where a PHMM is augmented with phylogenetic trees. In their approach, the evolutionary information is appended to the PHMM. They considered the dependency between sequences based on the fact that all the current sequences (external nodes in the guide tree or phylogenetic tree) are dependent upon their ancestral sequences. Based on their idea, there is no dependency between two current sequences.

But in our approach, the dependency between two current sequences based on the similarity between them can be appended to the PHMM. Based on the fact that with a MSA, the sequences are biologically related, we can use the MSA to find the areas of similarity between two current sequences. So, the MSA is used for consideration of the one-by-one dependency between observations. In other words, the dependency between corresponding amino acid located above the residue and the residue can be combined with the PHMM. Therefore the new parameters of PHMM called similarity emission (SE) probabilities are created and should be estimated.

It should be noted that the similarity emission probabilities are estimated from the MSA and then combined with the common emission probabilities estimated from Baum-Welch algorithm to generalize the Baum-Welch algorithm. In other words, both aligned and unaligned sequences are used to generalize the Baum-Welch algorithm: aligned sequences for estimation of the similarity emission probabilities and unaligned sequences for estimation of the common emission and transition probabilities.

In this paper, we first construct a PHMM. Then using a MSA, we model the similarity emission (SE) matrix for consideration of the similarity information and generalize the Baum-Welch algorithm. We finally compare the results of applying the similarity emission to the Baum-Welch algorithm with the results of the commonly used emission for sequence alignment. For this purpose we use real data from the top twenty protein families in the Pfam database [8].

## Materials and Methods

### 2.1 The PHMM

The profile hidden Markov model (PHMM) is a useful method to determine distantly related proteins by sequence comparison [3]. The PHMM is a linear structure of three states named; Match (M), Delete (D), and Insert (I). Therefore we need to decide how many states exist in a PHMM. In other words, how many match states do we have in a family? Here we assume that K is the number of match states in the PHMM. A commonly used rule is to set K equal to the number of columns of the MSA including more than half of the amino acid characters. Note that the number of match states is related to the length of the MSA [9]. So, the total number of M, D and I states is 3K. Begin and End states which emit no output symbols are introduced as dummy states [9]. Since there is an Insert state for each transition, there should be a transition from Begin called 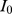. Therefore the total number of states is 3K+3. Twenty amino acids are observed from Match and Insert states. Delete, Begin and End states are silent states because they do not emit any symbols.

**Figure 1:**
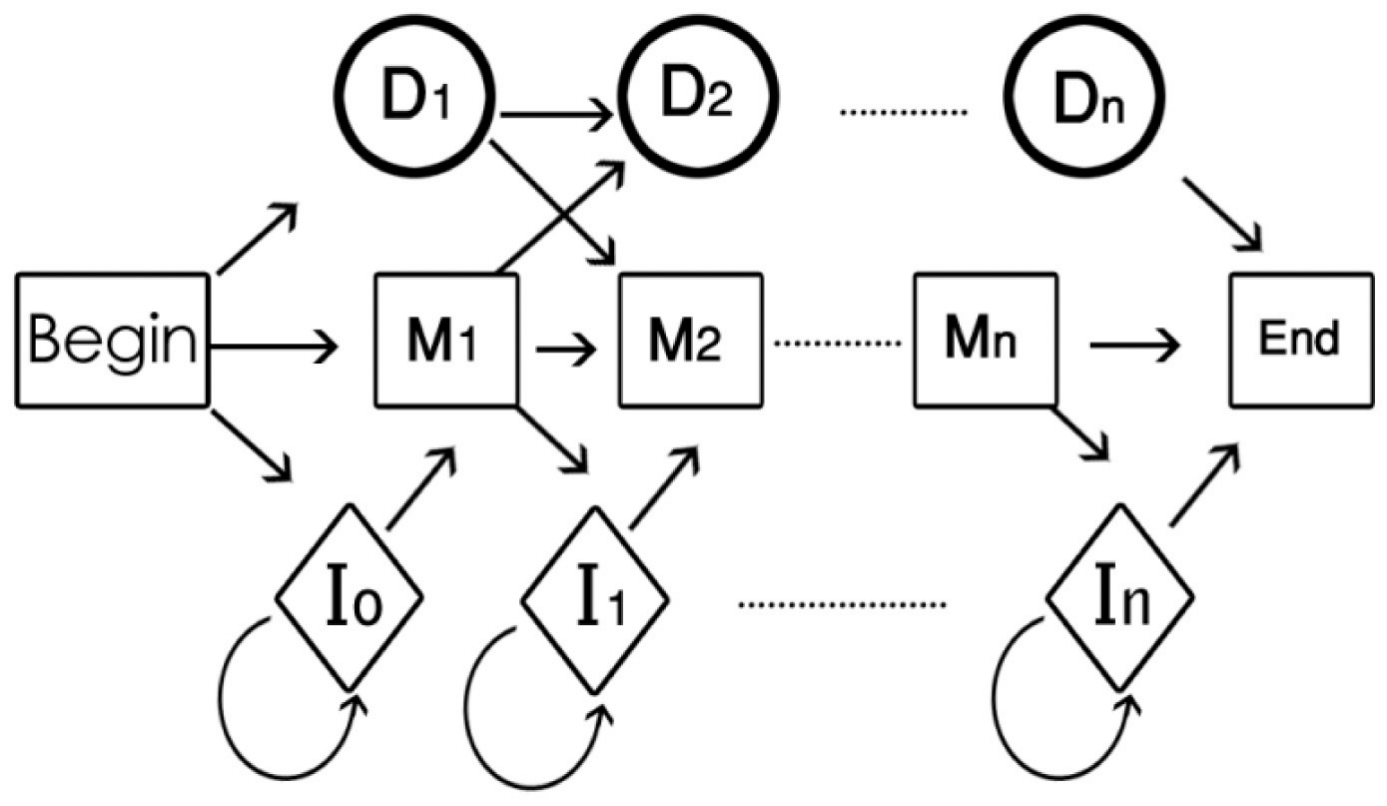
Plan 7 Construction.

Following Durbin [10], we estimate the transition probabilities, A, and the emission probabilities, B, using the plan7 construction (Figure 1). Unlike the original Krogh/Haussler and HMMER model architecture, Plan 7 has no D→I or I→D transitions. This reduction from 9 to 7 transitions per node in the main model is the origin of the codename Plan 7. Note that the transition probability 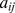 is the probability of moving from state 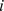 to state 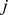 i.e.
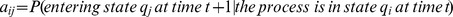
and emission probability 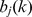 is the probability of observation 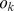 being emitted from state 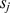 i.e.
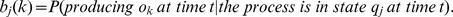
Parameters of a PHMM (transition probability matrix 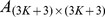, and emission probability matrix 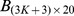) can be estimated using the Baum-Welch algorithm.

### 2.2 Considering The Similarity Between Sequences in the Baum-Welch Algorithm

The sequences appearing in the final multiple sequence alignment are written based on their similarity [10]. So, in a PHMM, the one-by-one dependency between corresponding amino acids of two current sequences can be considered. Therefore, we propose a model which considers the effect of the similarity information (the dependency between observation) as well as the effect of the hidden state on the previous state of an amino acid in a PHMM. For consideration of the similarity information, we introduce a similarity emission probability matrix based on the multiple sequence alignment. This matrix illustrates the similarity dependencies among the observations. Following the MSA, we assume that protein sequences consisting of 21 observations (20 amino acids and one gap) have been placed on a regular lattice. In other words, each observation is arranged as a site and a matrix with R rows and L columns (length of sequences) is obtained. This matrix is called the MSA matrix, in which the site position above the s = (r, c) is denoted by (r -1, c). Hence, we assume each site on the lattice has a dependency with the corresponding residue located at the above position. This scheme is a special case of the discrete state hidden Markov random field (HMRF) with 2-point cliques (Table 1). Note that the adjective ‘hidden’ refers to the states. The ingredients of this model are as follows:

**Table 1:** An example of the dependency between corresponding residues.

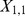	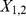	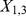
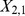	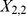	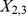
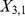	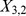 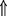	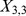
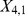	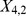	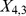

1. S: a set of lattice points
2. s: a lattice point, 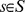, 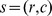, 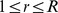, 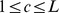

3. Emissions 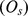: an observation at point s
4. Hidden states 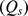: the hidden state at point s

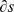
: the neighboring point of s (in this work, it is the above position of an amino acid)
6. Transition probabilities on the lattice: a matrix 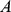 with following entries:
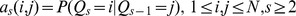
where, 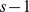 is a lattice point at previous state of s and 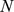 is the total number of hidden states.
7. Emission probabilities on the lattice: a matrix 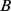 with the following entries:
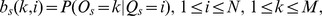
where 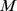 is a set of symbols that may be observed.
8. Emission probabilities on the lattice based on the above position: a matrix 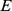 with the following entries representing the probabilities of the above position of an observation on the lattice:
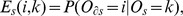
where 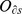 is an observation (amino acid or gap) at the above position.
9. Initial value: the probability of starting state at 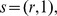 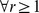:
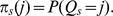

The likelihood of the parameters (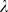) given the observations is:
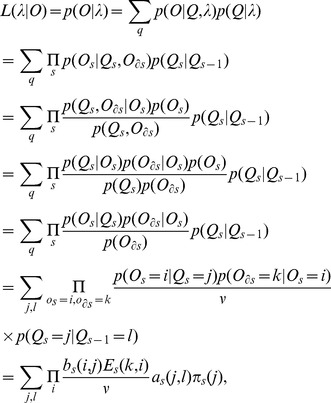
(1)where 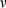 is a constant equal to 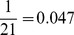. It should be noted that in Equation (1), the 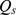 and 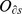 are independent, because 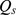 emits only 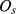. Based on Equation (1), we wish to find 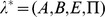, where 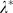 = 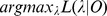. The entries of matrices 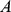, 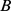, and the vector 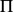 will be estimated through the following steps [11]:

1. Define auxiliary forward variable 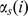 which is the probability of the partial observation sequence 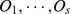 at lattice points 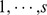 when it terminates at the state i:
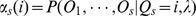

2. Define backward variable 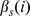 as the probability of the partial observation sequence 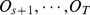 , given that the current state is i:
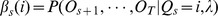

3. Calculate 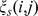 as the probability of being in state i at lattice point 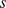 and in state j at lattice point 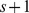, given observations and model:
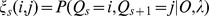
This is the same as,
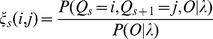
Using forward and backward variables this can be expressed as,
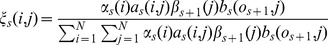

4. Define variable 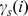 as the probability of being in state i at lattice point 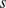, given the observations and model:
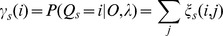
In forward and backward variables this can be expressed by,
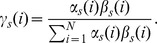
Now it is possible to use the Baum-Welch algorithm to maximize the quantity, 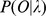. The estimation of parameters based on iterative calculation can be obtained by the following expressions:
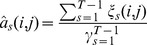


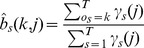


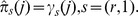
Hence, we have the matrices of parameter estimation 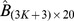, 
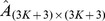
, and vector 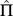.

Since the matrix 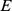 is a type of emission probability matrix, it should have the same size as the matrix 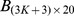. The estimation method for the matrix 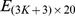 is as follows:

In the MSA matrix, the frequencies of ordered pairs of 20 amino acids and the gap i.e. 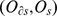 in each column are determined. It should be noted that 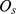 represents the amino acids (20 types) at lattice point s = 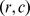 and 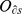 is the amino acids or one gap (21 types) located above 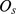. In other words, for a given amino acid 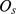, the position 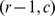 can be filled with any of 20 types of amino acids or the gap. hence, we can imagine of having a 420 (20
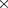
21) by 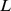 frequency matrix. After dividing these frequencies by the sum of frequencies in each column, the probabilities are estimated as follows:
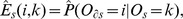


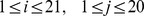
for which 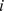 and 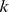 are amino acids or the gap and 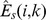 is the conditional probability of 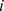 given 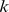 at lattice point s. This procedure produces the matrix 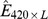.

In each column of matrix 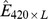, for every set of 21 probabilities, the highest probability is chosen. In other words, the highest probability for a given amino acid 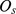 in the position 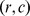 should be chosen. Then the matrix 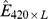 is reduced to a new matrix with 20 rows and 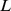 columns 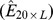. After transposing the matrix 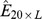, the matrix 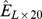 is obtained.

We assume that the 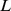 rows consist of Match and Insert states in which each Insert state can be repeated on its own several times. Using this assumption, we determine the Match states in 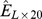 corresponding to Match states in 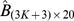. Note that there are 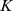 Match states. In addition, the average values of the rows between each of two Match states in 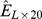 are considered as Insert states. So, the matrix 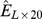 is changed to the matrix 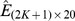 with 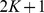 Match and Insert states in a row. The Delete states are included by adding zeros to the rows of 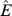, so that 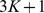 states are obtained.

Since the Begin and the End states are silent and do not emit any symbols, the two rows with zero number can be added at the beginning and the end of matrix 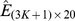. Consequently the matrix 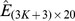 is obtained. This matrix is the estimation of the matrix {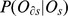}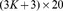.

### 2.3 Similarity Emission Matrix

The Baum-Welch algorithm defines an iterative procedure for estimating the parameters. It computes maximum likelihood estimators for the unknown parameters given observation [11]. Since the Baum-Welch algorithm finds local optima, it is important to choose initial parameters carefully. In this paper we perform the algorithm with different initial parameters in such a way that the transition probabilities into Match states are larger than transition probabilities into other states. In order to improve the prediction accuracy of assigning sequences to protein families, we consider both emission probability matrices 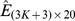 and 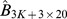. We generalize the Baum-Welch algorithm by integrating the both emission probability matrices 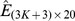 and 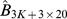 called similarity emission matrix 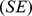. In what follows, we give the details:

1. Count the frequencies of ordered pairs of 20 amino acids and the gap, i.e., 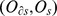 in each column of the MSA matrix
2. Calculate the probability matrix 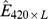 of ordered pairs by dividing frequencies by the sum of frequencies in each column with elements:
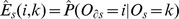

3. Choose the highest probability for each set of twenty one probabilities of each column of matrix 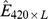, to obtain the matrix 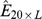

4. Transpose the matrix 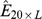 to obtain the matrix 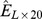

5. Write directly the values of Match states of 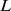 rows and the average values of Insert states between two Match states of the matrix 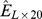 to obtain the matrix 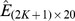. It should be noted the Match and Insert States will be obtained by using the multiple sequence alignment.
6. Add zero rows after each Match and Insert states to the 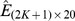 and also two zero rows as Begin and End states to obtain the matrix 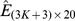

7. Use Hadamard product that is the entry-wise product of 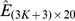 and 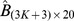 and then divide the entries by 0.047 to get the estimated similarity emission 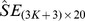 with the following entries:
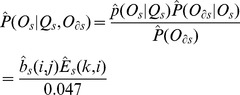
(2)

### 2.4 Data Preparation

The Pfam is a well known database of protein families [8]. It is widely used to align new protein sequences to the known proteins of a given family. There are two components in Pfam: Pfam-A and Pfam-B. The entries of Pfam-A have high quality. As shown in Table 2, we use twenty families of Pfam-A for assigning the protein sequences to these families. In this paper due to computational challenges and round-off errors in estimating parameters, we selected just twenty protein families from Pfam database which called top twenty HMM.

**Table 2:** Top twenty protein families in pfam database.

profile	Number of sequence	
	Seed	Full
ABC tran	60	163029
RVT 1	155	126258
COX1	94	118265
GP120	24	105452
WD40	1842	101999
RVP	50	93675
zf-C2H2	195	88330
Response_reg	57	75322
Cytochorm B N	92	70463
HA TPase c	662	70410
BPD transp 1	81	70027
MFS_1	196	69503
Oxidored q1	33	60333
Pkinase	54	56691
Cytochrom_B_C	114	51006
RVT_thumb	41	50191
Adh short	230	50144
Acetyltransf 1	243	46279
Helicase_C	491	42435
HTH1	1556	41545

## Results and Discussion

To assess the performance of our method, ten sequences from each of the top twenty families are randomly removed. These ten removed sequences in each family are used as test sequences, while the other sequences form the training set. We repeat this procedure ten times. Since some of the protein families contain few proteins (likeGP120 and Oxidored q1), we choose just ten samples. Therefore, each time we have selected 200 sequences. In total 2000 sequences are randomly removed. Then we estimate the transition matrix 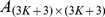, emission matrices 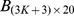 and 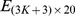 for each protein family. Given top twenty protein families, the score of each removed sequence belonging to each family are computed and compared. To score a sequence and assign it to one of the top twenty families, we use the logarithm of the probability score. It is defined by
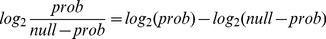
(3)where prob is the probability of sequence based on parameter estimation and null-prob is equal to 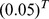 where 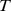 is the length of sequence. Since there are twenty amino acids, the probability of random occurrence of each of them is 0.05. Hence, for a sequence of 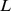 amino acids, the probability of random occurrence is 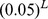.

In this paper, due to computational challenges and round-off errors in estimating probabilities of 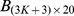 and 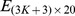, we have employed logarithm transformation instead of the direct multiplication of these probabilities:
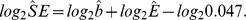
The mean and standard error of the numbers of correctly assigned proteins to the top twenty protein families are shown in Table 3. Based on the results shown in Table 3, the assignment of sequences to the protein families using the 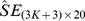 is considerably improved. For all protein families, more than half of the sequences are assigned correctly. In the task of assigning protein sequences, measuring the specificity is also important to prevent false positive prediction. Specificity is a statistical measure of the performance of a classification test, also known in statistics as classification function. Specificity measures the proportion of negatives which are correctly identified. This measure is closely related to the concepts of type II errors in testing a statistical hypothesis . Specificity relates to the ability of the test to identify negative results. This can also be written as:
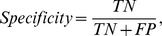
where, 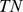 is the number of True Negative and 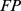 is the number of False Positive. In other words, specificity means how many of the true negatives are detected? Ideally, suitable method should have high specificity or a perfect predictor would be described as 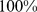 specificity. The specificity on average is about 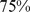 and 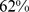 using similarity emission and common emission model respectively. In addition to the correct assignment, the mean of the standard assigning scores, based on the matrix 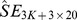 in most families are more than those obtained by the matrix 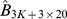 (Table 4). The results presented in this paper show that considering a model which incorporates the similarity information of the corresponding amino acid located above a residue in a protein family will result in a notable improvement in assignment task. It should be noted that based on the MSA implemented by ClustalW, one-by-one dependencies between corresponding amino acids of two current sequences that model the similarity between them can be appended to the PHMM. In other words, we combine the similarity emission matrix obtained form the aligned sequences and common emission matrix obtained from the unaligned sequences to generalize the Baum-Welch algorithm.

**Table 3:** The mean and standard error of the numbers of correctly assigned sequences.

profile	Mean		Standard Error	
	Using 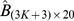	Using 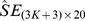	Using 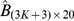	Using 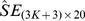
ABC_tran	6.200	9.100	0.805	0.482
RVT 1	9.102	9.723	0.588	0.531
COX1	5.529	9.34	0.534	0.482
GP120	9.034	9.980	0.460	0.405
WD40	7.515	8.601	0.672	0.520
RVP	6.129	8.802	0.801	0.672
zf-C2H2	1.980	9.001	0.534	0.578
Response_reg	8.456	8.991	0.555	0.612
Cytochorm B N	7.800	8.901	0.850	0.601
HA TPase c	7.098	9.992	0.640	0.504
BPD transp 1	7.091	8.002	0.605	0.604
MFS_1	8.409	8.997	0.583	0.538
Oxidored q1	8.001	8.973	0.593	0.471
Pkinase	2.009	8.623	0.981	0.812
Cytochrom_B_C	8.032	9.010	0.524	0.503
RVTthumb	6.839	8.902	0.835	0.561
Adh short	6.998	8.572	0.984	0.607
Acetyltransf 1	6.504	9.760	0.551	0.504
HelicaseC	7.228	8.423	0.682	0.634
HTH_1	1.734	7.991	0.609	0.684

**Table 4:** The mean and standard error of the standard scores of assigning sequences to each protein family based on the emission matrix 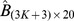 and similarity emission matrix 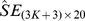.

profile	Mean		Standard Error	
	Using 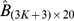	Using 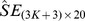	Using 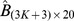	Using 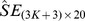
ABC_tran	−0.834	−0.503	0.054	0.043
RVT 1	−0.546	−0.504	0.213	0.113
COX1	0.789	0.881	0.085	0.054
GP120	0.115	0.234	0.085	0.079
WD40	0.356	0.487	0.076	0.065
RVP	0.244	0.307	0.082	0.058
zf-C2H2	−0.567	−0.523	0.048	0.043
Response_reg	−0.775	−0.709	0.061	0.062
Cytochorm B N	2.143	3.4452	0.233	0.231
HA TPase c	1.814	3.651	0.202	0.200
BPD transp 1	0.807	0.718	0.069	0.058
MFS_1	−0.213	−0.035	0.082	0.044
Oxidored q1	−0.403	−0.352	0.050	0.078
Pkinase	−0.046	0.567	0.070	0.065
Cytochrom_B_C	−0.749	−0.757	0.089	0.055
RVT_thumb	0.005	0.142	0.057	0.021
Adh short	−0.550	−0.523	0.079	0.078
Acetyltransf 1	0.453	0.501	0.053	0.059
HelicaseC	0.478	0.501	0.078	0.076
HTH_1	0.640	0.703	0.070	0.052
